# Bioinformatics and *in-silico* findings reveal medical features and pharmacological targets of biochanin A against colorectal cancer and COVID-19

**DOI:** 10.1080/21655979.2021.2005876

**Published:** 2021-12-23

**Authors:** Jingru Qin, Chao Guo, Lu Yang, Xiao Liang, Aijun Jiao, Keng Po Lai, Bin Yang

**Affiliations:** a College of Pharmacy, Guangxi Medical University, Nanning, Guangxi, PR China; b Department of Pharmacy, Guigang City People’s Hospital, the Eighth Affiliated Hospital of Guangxi Medical University, Guigang, Guangxi, PR China; c Laboratory of Environmental Pollution and Integrative Omics, Guilin Medical University, Guilin, PR China

**Keywords:** Colorectal cancer, COVID-19, biochanin a, bioinformatics, biological function, pharmaceutical target

## Abstract

Severe mortality due to the COVID-19 pandemic resulted from the lack of effective treatment. Although COVID-19 vaccines are available, their side effects have become a challenge for clinical use in patients with chronic diseases, especially cancer patients. In the current report, we applied network pharmacology and systematic bioinformatics to explore the use of biochanin A in patients with colorectal cancer (CRC) and COVID-19 infection. Using the network pharmacology approach, we identified two clusters of genes involved in immune response (*IL1A, IL2*, and *IL6R*) and cell proliferation (*CCND1, PPARG*, and *EGFR*) mediated by biochanin A in CRC/COVID-19 condition. The functional analysis of these two gene clusters further illustrated the effects of biochanin A on interleukin-6 production and cytokine-cytokine receptor interaction in CRC/COVID-19 pathology. In addition, pathway analysis demonstrated the control of PI3K-Akt and JAK-STAT signaling pathways by biochanin A in the treatment of CRC/COVID-19. The findings of this study provide a therapeutic option for combination therapy against COVID-19 infection in CRC patients.

## Introduction

COVID-19 is a global pandemic disease. There have been 134,308,070 confirmed cases of COVID-19 and 2,907,944 deaths reported to the WHO worldwide (as of 7 April 2021). Although different vaccines against COVID-19 are available, patients with chronic diseases, especially cancer patients infected by COVID-19, are at a high risk of death [1]. Colorectal cancer (CRC) is the second most common cause of cancer-related death in the United States. In 2020, approximately 147,950 individuals were diagnosed with CRC, and 53,200 CRC patients died from the disease [2]. COVID-19 delayed elective CRC surgery in several cases, leading to poorer overall survival (OS) and disease-free survival (DFS) [3]. More importantly, the therapeutic methods for CRC patients with COVID-19 are different from the usual methods. This is because aggressive treatment of cancer may further lower immunity, making CRC patients more susceptible to COVID-19 [4]. Therefore, there is an urgent need to discover treatment alternatives for patients with CRC and COVID-19. Biochanin A, an O-methylated isoflavone, is a natural organic compound in the flavonoid class. It is considered a dietary anticancer prophylactic with antiapoptotic properties [5]. Biochanin A promotes apoptosis and cell cycle arrest in cancer cells [6,7]. Reportedly, biochanin A can be used in combination with radiation treatments, because it enhances radiotoxicity in colon tumor cells [8]. Furthermore, accumulating reports have demonstrated that biochanin A can protect the lung against toxicity and injury[9], and that it may interfere with SARS-CoV-2 attachment to the host cell. In addition, biochanin A suppresses airway hyperresponsiveness by inhibiting phosphodiesterase [10].

In this study, we hypothesized that biochanin A is a potential drug for treating CRC patients with COVID-19. By using network pharmacology and bioinformatics, we aimed to determine pharmaceutical targets of biochanin A for treating CRC patients with COVID-19. We have identified gene clusters associated with CRC, COVID-19, and biochanin A. Bioinformatic analysis further showed the involvement of six core targets of biochanin A in the treatment of CRC/COVID-19, including *EGFR, CCND1, IL2, IL1A, IL6R*, and *PPARG*. Gene ontology (GO) and Kyoto Encyclopedia of Genes and Genomes (KEGG) pathway analysis suggested that the therapeutic effect of biochanin A was due to the regulation of interleukin-6 production, regulation of inflammatory response, and alteration in different cell signaling pathways (such as cytokine-cytokine receptor interaction, cytokine-mediated signaling pathway, PI3K-Akt signaling pathway, and Jak-STAT signaling pathway). This study is the first to reveal the clinical use and pharmaceutical targets of biochanin A for the treatment of CRC patients with COVID-19. The goal of this study was to identify a new drug for combination therapy and to improve the survival rate of CRC patients infected with COVID-19.

## Materials and methods

### Searching CRC- and COVID-19-associated genes

To identify CRC/COVID-19-associated genes, the transcriptomic profile of CRC patients was obtained from The Cancer Genome Atlas (TCGA) database (https://portal.gdc.cancer.gov/) on 29 August 2020. For the COVID-19-associated genes, different databases including the Genecards database [11,12], Online Mendelian Inheritance in Man (OMIM) database (https://omim.org/), and National Center for Biotechnology Information (NCBI) (https://www.ncbi.nlm.nih.gov/) were screened. Then, the CRC- and COVID-19-associated genes were overlaid to determine the predisposing genes of CRC/COVID-19.

### Identification of pharmaceutic targets of biochanin A for treating CRC/COVID-19

The chemical structure of biochanin A was obtained from the Traditional Chinese Medicine Systems Pharmacology Database and Analysis Platform (TCMSP) [13]. The pharmacological targets of biochanin A were determined using online accessible tools and databases including Swiss Target Prediction [14], SuperPred [15], TargetNet [16], Batman [17], and Drugbank [18,19]. The identified genes were corrected with the UniProt database using the Swiss-Prot human database (www.uniprot.org/). The biochanin A-associated genes were then overlapped with CRC/COVID-19-associated genes. The CRC/COVID-19/biochanin A overlapping genes were subjected to the STRING database (version 11.0) revision to obtain a network of protein-protein interactions (PPI) [20]. The network analyzer setting from Cytoscape was used to analyze the topological parameters. Core targets were collected according to the degree-algorithm values [21,22].

### Functional characterization of biochanin A against CRC/COVID-19

Gene Ontology (GO) biological process (BP) and Kyoto Encyclopedia of Genes and Genomes (KEGG) pathways enrichment analysis using R language packages including ‘ClusterProfiler,’ ‘ReactomePA,’ ‘org.Hs.eg.Db,’ and ‘GOplot’ was conducted to determine the alteration of biological processes and cell signaling pathways mediated by biochanin A against CRC/COVID-19 [23]. The Cytoscape plot (3.7.1 version) was used to visualize the correlation of biological processes and pathways of biochanin A against CRC/COVID-19 [21].

## Results

As COVID-19 is a threat to CRC patients, this study aimed to discover a new drug to increase the survival rate of CRC patients with COVID-19. We hypothesized that biochanin A, a potential drug, may help patients. By using network pharmacology and systematic bioinformatic analysis, we intended to delineate pharmaceutical targets and molecular mechanisms underlying the potential role of biochanin A against COVID-19 and CRC.

### Identification of CRC/COVID-19-associated genes

**Figure 1.**
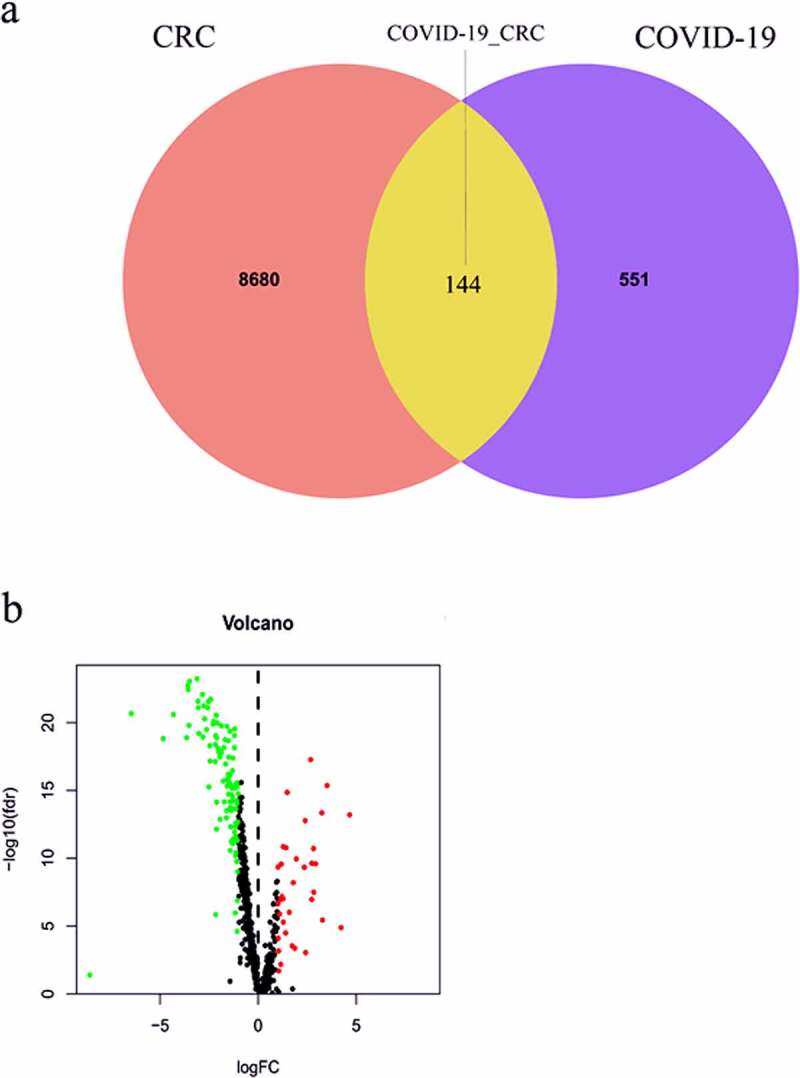
**Identification of CRC/COVID-19-associated genes**. (a) The Venn diagram showed the number of intersecting genes in CRC/COVID-19. (b) Volcano plot showed the expression level of differential expressed CRC/COVID-19-associated genes in CRC. The genes with |log 2 (fold change)| > 1 and -log 10 (FDR) > 1.3 were considered as differential expressed genes

To determine the CRC-associated genes, we searched the transcriptome profiles of CRC patients in the Cancer Genome Atlas (TCGA) database. The search identified 8,824 CRC-associated genes (Figure 1a). For the identification of COVID-19-associated genes, we harvested the data from the Genecards, OMIM, and NCBI databases. A total of 695 COVID-19-associated genes were identified (Figure 1a). When we compared the CRC- and COVID-19-associated genes, we found that they shared 144 genes (Figure 1a). Of these, 36 upregulated and 108 downregulated genes were observed in CRC patients (Figure 1b).

### Identification of pharmaceutic targets and mechanisms of biochanin A for treating COVID-19 and CRC

**Figure 2.**
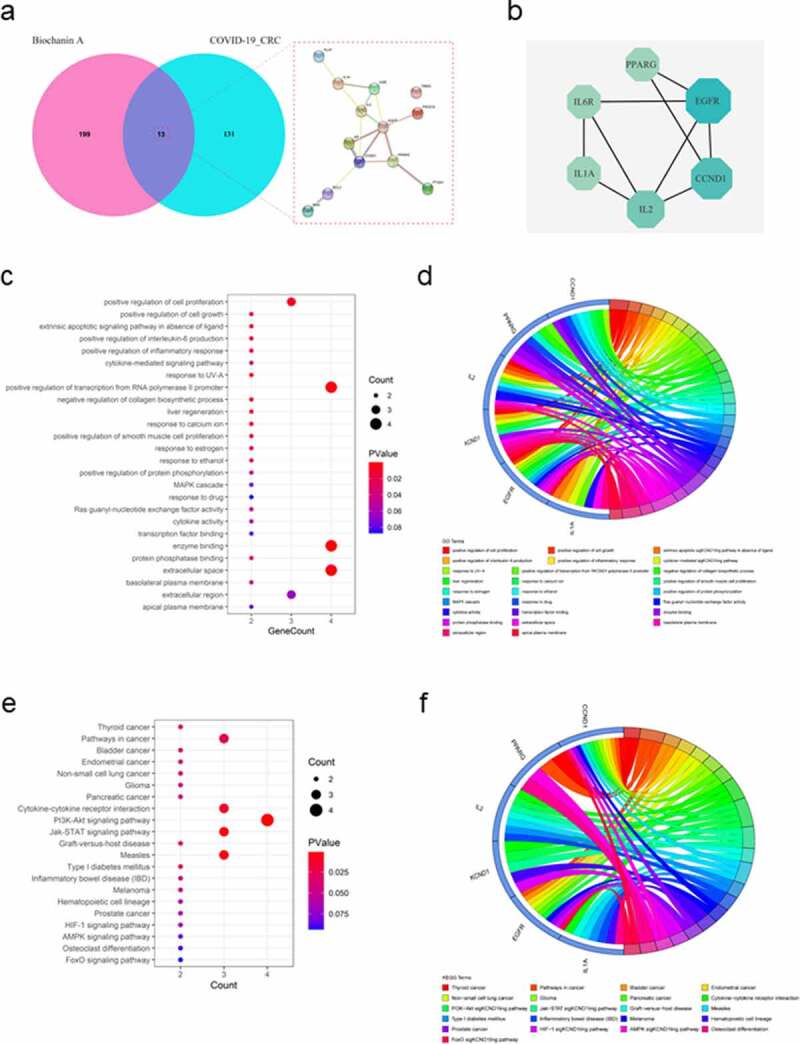
**Functional characterization of biochanin A/CRC/COVID-19-associated genes**. (a) The Venn diagram showed the number of intersecting genes in biochanin A/CRC/COVID-19. (b) Protein-protein interaction analysis of biochanin A/CRC/COVID-19-interacting genes using STRING tool. (c) Gene ontology enrichment analysis highlighted the biological processes and molecular functions controlled by biochanin A/CRC/COVID-19-associated genes. The size of each dot represents the number of genes. The color intensity of the dot represents the significance of the processes. (d) Circos plot shows the involvement of genes in the enriched biological processes. (e) Kyoto Encyclopedia of Genes and Genomes analysis highlighted the cell signaling pathways mediated by biochanin A/CRC/COVID-19-associated genes. The size of dots represents the number of genes. The color intensity of the dot represents the significance of the pathways. (f) Circos plot showed the involvement of genes in the enriched signaling pathways

**Figure 3.**
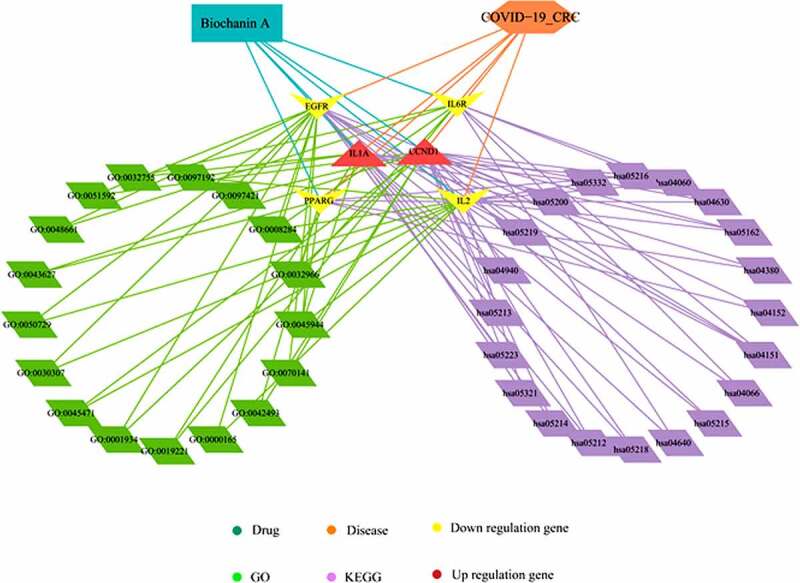
**Biological processes and pathways mediated by biochanin A/CRC/COVID-19-associated genes**. By using the Cytoscape tool, the biological processes have been highlighted in green and the pathways in purple. The brown shape represents upregulated genes and the yellow shape represents downregulated genes

To determine the possible pharmacological role of biochanin A, we harvested biochanin A-associated genes from different databases including Swiss Target Prediction, SuperPred, TargetNet, Batman, and Drugbank. We identified 212 biochanin A-associated genes (Figure 2a). We then overlaid the biochanin A-associated genes with CRC/COVID-19-associated genes to identify possible targets of biochanin A for treating CRC/COVID-19. Our results showed 13 potential targets of biochanin A; the molecular interaction network analysis using Cytoscape further highlighted the involvement of six core targets of biochanin A, *EGFR, CCND1, IL2, IL1A, IL6R*, and *PPARG*, for the treatment of CRC/COVID-19 (Figure 2b). To further understand the biological roles and cell signaling pathways mediated by these targets, GO and KEGG pathway enrichment analyses of the six core targets were performed. GO analysis revealed that biochanin A altered the biological processes related to cell proliferation, cell growth, and apoptosis (Figure 2(c, d)). In addition, biochanin A modulates the immune and inflammatory responses such as interleukin-6 production and cytokine-mediated signaling pathways (Figure 2(c,d)). For the molecular function, the result suggested the effect of biochanin A on Ras guanyl-nucleotide exchange factor activity, transcription factor binding, and cytokine activity (Figure 2(c,d)). The KEGG pathway analysis further highlighted several cancer types including thyroid cancer, bladder cancer, endometrial cancer, non-small cell lung cancer, glioma, and pancreatic cancer (Figure 2(e,f)). In addition, cytokine-cytokine receptor interaction, PI3K-Akt signaling pathway, and Jak-STAT signaling pathway were found to be targeted by biochanin A (Figure 2(e,f)). Taken together, our results suggest that biochanin A could target genes involved in different biological functions and cell signaling pathways in CRC/COVID-19 patients (Figure 3).

### Binding of biochanin A to EGFR

**Figure 4.**
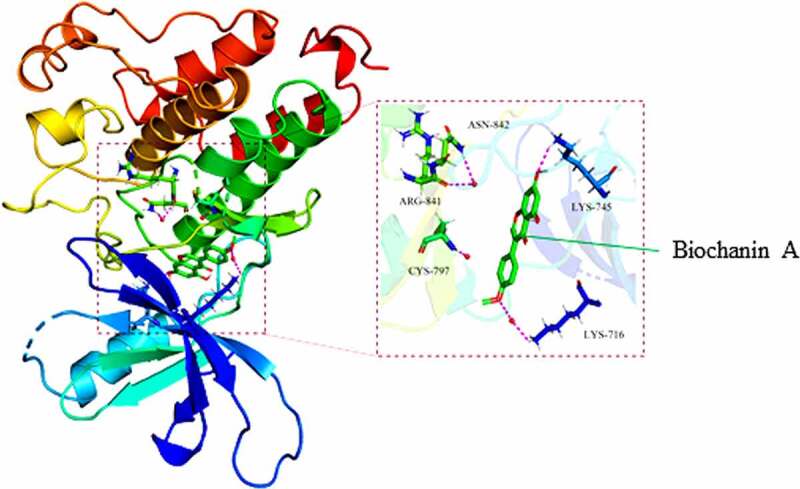
The direct binding of biochanin A to epidermal growth factor receptor (EGFR)

In the last part of the study, we aimed to determine the possible direct binding of biochanin A to its targets. Among the identified targets, we only found a direct interaction between biochanin A and its target EGFR (Figure 4). The protein structure of EGFR was obtained from the Protein Data Bank (PDB) database (PDB ID: 5UGC; http://www.rcsb.org/structure/5UGC). The active cavity box of EGFR was set as follows (center: x, y, z: −13.754, 13.949, −25.235; size: x, y, z: 11.25, 11.25, 11.25) using the programs Autodock and Visual Molecular Dynamics. The structure of EGFR was visualized using PyMOL (version 2.3) (Figure 4). Our results showed that biochanin A formed a hydrogen bond with the amino acid residues LYS-716 (2.5 Å) of EGFR through hydrolysis (Figure 4), suggesting a direct interaction between biochanin A and the EGFR protein.

The chemical structure of biochanin A was obtained from the Traditional Chinese Medicine Systems Pharmacology Database and Analysis Platform. The protein structure of EGFR was obtained from the Protein Data Bank (PDB) database. AutoDock and Visual Molecular Dynamics identified that hydrogen bonds are formed between the amino acid residues LYS-716 (2.5 Å) of EGFR and biochanin A.

## Discussion

COVID-19 has caused 2,907,944 deaths worldwide. COVID-19 vaccines are available but their protective efficiency varies. More importantly, the side effects of the vaccine make it difficult to apply to patients with chronic diseases, especially cancer patients. One of the most serious outcomes is a high mortality. In this study, we aimed to apply network pharmacology analysis followed by systematic bioinformatics analysis to uncover the possible use of biochanin A for treating CRC/COVID-19. ‘Cytokine storms’ are associated with the severity of COVID-19 in patients [24,25]. Therefore, the identification of drugs that target immune responses may help to relieve the symptoms of COVID-19. In our analysis, we identified two target gene clusters of biochanin A including inflammatory genes (*IL1A, IL2*, and *IL6R*) and cell proliferation genes (*CCND1, PPARG*, and *EGFR*) relevant to the treatment of CRC/COVID-19.

Interleukin 1 alpha (IL1A) causes inflammation and sepsis. A transcriptomic study of COVID-19 patients showed the induction of IL1A in nasopharyngeal samples [26]. The other identified biochanin A targeted gene IL2 is an important cytokine that regulates the activities of leukocytes, and it was reported to be elevated in patients with COVID-19 [27]. A cell signaling study in COVID-19 suggested that the JAK/STAT signaling pathway is one of the major pathways controlling IL2 [28]. Our results also highlighted that the interleukin 6 receptor (IL6R) was the target of biochanin A. As a receptor of IL6, it is one of the cytokines induced in patients with COVID-19. IL6, a pro-inflammatory cytokine, plays a crucial role in inflammatory responses, autoimmune diseases, and viral infections [29]. IL6 signaling has been reported to be important for the acute-phase immunological response and to promote anti-inflammatory activities in the pulmonary system [30]. In terms of cancer research, different case-control studies have suggested the association of IL1A, IL2, and IL6 with CRC. For instance, IL1A polymorphisms were found to be a risk factor for colorectal cancer in the Chinese Han population [31]. Another genetic variant analysis of CRC patients demonstrated that high expression level of IL2 was related to risk and survival in patients with CRC [32], suggesting the importance of IL1A and IL2 in the tumorigenesis of CRC. In addition, increased IL-6 expression is associated with advanced disease stage and decreased survival in CRC patients. It promotes tumor cell proliferation and inhibits apoptosis.

In addition to immune-responsive genes, our findings also highlighted a cluster of cell proliferation genes targeted by biochanin A. Cyclin D1 (CCND1), the key regulator of CDK kinases in the cell cycle, is a cell-cycle regulatory molecule commonly induced at the sites of inflammation in lung and colon tissues. CCND1 has been reported to play a role in progressive pulmonary inflammation and fibrosis [33] and lung innate immunity [34]. In addition, CCND1 is the downstream effector of β‑catenin facilitating the control of chronic inflammation and organ fibrosis [35]. In the docking analysis, we further demonstrated the possible direct binding of biochanin A to its target EGFR. Epidermal growth factor receptor (EGFR), a transmembrane receptor of extracellular protein ligands, is deregulated in many cancer types [36,37]. EGFR is an important therapeutic target for cancer treatment because of its multifunctional role in tumor malignancy [38]. It has been reported that EGFR is also a target of COVID-19 treatment. For instance, a meta-analysis showed that EGFR is involved in pathological effects on the COVID-19 inflammatory process [39]. A drug discovery study demonstrated that the anti-COVID-19 action of puerarin was associated with the suppression of oxidative stress and inflammatory cascades through the induction of EGFR [40]. In addition to the pharmaceutical targets, the mechanisms underlying the effect of biochanin A were discovered using pathway analysis. Our results highlighted some important pathways such as cytokine-cytokine receptor interaction, PI3K-Akt signaling pathway, and JAK-STAT signaling pathway targeted by biochanin A through the control of gene clusters (*CCND1, IL1A, IL2, KCND1*, and *EGFR*). These pathways have been reported to be associated with immune responses in the lung and the carcinogenicity of CRC [41,42]. Cytokine-cytokine receptor interaction is responsible for the regulation of the tumor microenvironment and tumor cells [43]. This pathway has been reported to be a key pathway involved in drug response in CRC cells [44]. The PI3K-Akt signaling pathway is involved in autophagy, leading to the regulation of the inflammatory response and progression of pulmonary fibrosis [45e]. In an asthmatic mouse model, the PI3K-Akt-mTOR pathway was found to inhibit the Beclin-1-PI3KC3 protein complex in lung tissues [46]. A few studies have suggested the role of the PI3K-Akt pathway in COVID-19. For instance, PI3K-Akt sustaining aerobic glycolysis inhibits the replication of MERS-CoV and, presumably, can inhibit that of COVID-19 as well [47]. In addition, COVID-19 can activate the PI3K-Akt signaling pathway and can play dual anti-inflammatory and carcinogenic roles [48]. The JAK-STAT signaling pathway that activates cytokines has been suggested as a target for preventing intense lung injury and increasing the chances of survival in COVID-19 patients [49]. More importantly, a genetic polymorphism study of COVID-19 patients showed the polymorphism locus near the Janus kinase (JAK) TYK2, the key for IFN, interleukin (IL)-12, and IL-23 signaling, and T helper (Th) 1/Th17 cell mediated antiviral immune responses, suggesting the critical role of the JAK-STAT signaling pathway in the development of cytokine storm in COVID-19 [28].

## Conclusions

This report uncovers pharmaceutical targets of biochanin A, including EGFR, CCND1, IL2, IL1A, IL6R, and PPARG, for the treatment of CRC/COVID-19. Additionally, the identification of its pharmacological functions (such as immune and inflammatory responses, cell proliferation, cell growth, and apoptosis) and its therapeutic mechanisms (involving cytokine-cytokine receptor interaction, PI3K-Akt signaling pathway, and Jak-STAT signaling pathway) may lead to treatment options for combined therapy against CRC/COVID-19. However, our findings are based on network pharmacology and bioinformatics, and clinical and biological studies are needed to validate our results.

## Data Availability

Uploaded as supplementary files.

## Abbreviations

CRC, colorectal cancer; COVID-19, coronavirus disease 2019; TCGA, The Cancer Genome Atlas; GO, Gene Ontology; BP, Biological process; KEGG, Kyoto Encyclopedia of Genes and Genomes; TCMSP: Traditional Chinese Medicine Systems Pharmacology Database and Analysis Platform.
